# lncRNA IGF2-AS Regulates Nucleotide Metabolism by Mediating HMGA1 to Promote Pyroptosis of Endothelial Progenitor Cells in Sepsis Patients

**DOI:** 10.1155/2022/9369035

**Published:** 2022-01-17

**Authors:** Guibin Liang, Menghao Zeng, Min Gao, Wei Xing, Xin Jin, Qianlu Wang, Longtian Deng, Hao Ou, Zhihui He

**Affiliations:** Department of Critical Care Medicine, The Third Xiangya Hospital, Central South University, Changsha 410013, China

## Abstract

**Background:**

Sepsis is one of the major causes of death worldwide, and its high mortality and pathological complexity hinder early accurate diagnosis. We aimed to investigate lncRNA IGF2-AS and HMGA1 effects on pyroptosis of endothelial progenitor cells (EPCs) in sepsis patients and the mechanisms involved.

**Methods:**

Blood samples from sepsis patients and healthy subjects were collected, and EPCs were isolated and identified. We constructed cell lines that knocked down lncRNA IGF2-AS, HMGA1, and TYMS. Furthermore, lncRNA IGF2-AS was overexpressed. Subsequently, dNTP treatment with different concentrations was performed to investigate lncRNA IGF2-AS and HMGA1 effects on pyroptosis of EPCs in sepsis patients. Finally, exosomes were isolated from bone marrow mesenchymal stem cells (MSCs) to detect lncRNA IGF2-AS expression, and the influence of MSC-derived exosomal lncRNA IGF2-AS on sepsis was preliminarily discussed.

**Results:**

Compared with Healthy group, lncRNA IGF2-AS, HMGA1, and TYMS were highly expressed in Sepsis group. Compared with si-NC group, si-lncRNA IGF2-AS group had increased proliferation ability, decreased pyroptosis, decreased HMGA1, RRM2, TK1, and TYMS expressions. lncRNA IGF2-AS played a regulatory role by binding HMGA1. Compared with si-NC group, the proliferation ability of si-HMGA1 group increased, pyroptosis decreased, and RRM2, TK1, and TYMS expressions also decreased. Compared with si-NC group, pyroptosis in si-TYMS group was reduced. In addition, HMGA1 was related and bound to TYMS. After overexpressing lncRNA IGF2-AS, dNTP level decreased, while the proliferation increased and pyroptosis decreased with higher concentration of dNTP. In addition, we found that EPCs took up MSC-exosomes. Compared with supernatant group, lncRNA IGF2-AS was expressed in exosomes group. Compared with EPCs group, EPCs+exosomes group had increased lncRNA IGF2-AS expression and increased pyroptosis.

**Conclusions:**

lncRNA IGF2-AS regulated nucleotide metabolism by mediating HMGA1 to promote pyroptosis of EPCs in sepsis patients. This study provided important clues for finding new therapeutic targets for sepsis.

## 1. Introduction

Sepsis is a life-threatening organ dysfunction caused by the host's dysregulated response to infection [1]. It is one of the major causes of death worldwide, and its high mortality and pathological complexity hinder early accurate diagnosis [2]. Clinically used blood markers C-reactive protein (CRP) and procalcitonin (PCT) are indicators of acute phase reactions, thus lacking specificity and providing limited diagnostic efficacy [3]. Sepsis is often accompanied by severe vascular endothelial injury and barrier dysfunction [4]. It has been shown that the endogenous repair mechanism of damaged vascular endothelium requires local proliferation of endothelial cells, but the process of reendothelialization and angiogenesis after the endothelial injury is affected by endothelial progenitor cells (EPCs) [5]. In addition, the number of EPCs is increased in sepsis patients and is related to survival [6]. Abundant evidence shows that sepsis is related to pyroptosis [7]. During sepsis, pyroptosis is required for defense against bacterial infection because appropriate pyroptosis can minimize tissue damage. However over-activated pyroptosis can lead to septic shock, multiple organ dysfunction, or an increased risk of secondary infection [8]. However the potential value of EPCs pyroptosis in the treatment of sepsis has not been explored.

Long noncoding RNAs (lncRNAs) are functional RNA fragments with more than 200 nucleotides in length, which have become important participants in the function and regulation level of almost all genes [9]. In recent years, lncRNAs have been confirmed to be involved in sepsis. It has been reported that lncRNA MALAT1 interacts with EZH2 to stimulate AKT-1 phosphorylation and reduces BRCA1 expression, thereby aggravating the progression of sepsis [10]. Previous study has shown that lncRNA RMRP can inhibit lipopolysaccharide-induced mitochondrial dysfunction and myocardial cell apoptosis in mice with sepsis through miR-1-5p/hsp70 axis [11]. However, there are still few reports on the molecular mechanisms related to lncRNA IGF2-AS in sepsis, and further exploration is needed. Exosomes are nano-sized vesicles that carry proteins, lipids, and nucleic acids to promote cellular communication [12]. Study has shown that the content and function of exosomes are changed during sepsis [13]. In addition, a variety of mediators carried by exosomes are crucial to the pathogenesis of sepsis-related organ dysfunction [14]. Exosomal lncRNA-p21 derived from mesenchymal stem cells (MSCs) has been reported to increase sirtuin 1 expression, thereby protecting MLE-12 cells from apoptosis during sepsis [15]. Therefore, the study of exosomal lncRNA IGF2-AS derived from MSCs in sepsis may provide ideas for treating sepsis.

High mobility group A (HMGA) is a non-histone chromatin protein that binds to DNA microgrooves, interacts with transcriptional mechanisms, and promotes DNA-oriented nucleation [16]. HMGA1 plays a role in gene regulation that promotes the systemic inflammatory response. It has been reported that blocking HMGA1-mediated pathways may improve the sepsis prognosis [17]. Bioinformatics analysis showed that lncRNA IGF2-AS had a targeted binding site with HMGA1. Therefore, we wanted to further investigate the roles of lncRNA IGF2-AS and HMGA1 in sepsis. Nucleotides are required for various biological processes and continuously synthesized de novo in all cells [18]. It has been reported that lncRNA NMR regulates tumor cell proliferation by regulating the YBX1-RRM2-TYMS-TK1 axis of nucleotide metabolism [19]. In sepsis, cAMP metabolism controls the activation and pyroptosis of caspase-11 inflammatory bodies [20]. However, there are few studies on nucleotide metabolism in sepsis. We try to investigate the influence of nucleotide metabolism in sepsis.

In this study, blood samples from sepsis patients and healthy subjects were collected, and EPCs were separated and identified. We took EPCs of sepsis patients as the research object and explored lncRNA IGF2-AS and HMGA1 effects on the pyroptosis of EPCs in patients with sepsis, and the mechanisms involved in it through in vitro cell experiments. In addition, we preliminarily discussed the influence of exosomal lncRNA IGF2-AS derived from bone marrow MSCs on sepsis. Our study provides important clues for finding new therapeutic targets for sepsis.

## 2. Materials and Methods

### 2.1. Clinical Samples

Twelve sepsis patients were from the Third Xiangya Hospital, Central South University. Twelve healthy subjects were selected as the control. Their peripheral blood was obtained in the Third Xiangya Hospital, Central South University. All sepsis patients have been confirmed by examination in the Third Xiangya Hospital, Central South University. The diagnostic criteria of sepsis refer to the International Guidelines for Management of Sepsis and Septic Shock: 2016 [21]. Healthy subjects did not have sepsis at the time of physical examination.  Table 1 showed the clinical characteristic of sepsis patients and healthy subjects. This study was approved by The IRB of Third Xiangya Hospital, Central South University (No.2020-S570). Written informed consent of each subject has been obtained for this study.

### 2.2. Isolation of EPCs

Under aseptic conditions, peripheral blood was collected from healthy physical examination subjects and sepsis patients for obtaining EPCs. Mononuclear cells were isolated by density gradient centrifugation. EGM-2 complete medium (CC-3162, LONZA) was used to culture in an incubator at 37°C, 5% CO_2_, and saturated humidity. When the cell fusion rate reached 70-80%, cells were digested and collected with 0.25% trypsinase (including 0.02% EDTA) to prepare cell suspension. It was then centrifuged at 1000 rmp for 5 min, and the supernatant was discarded. Precipitation was resuspended with EGM-2 complete medium, and subculture was carried out in a ratio of 1 : 2 or 1 : 3. They were divided into the Healthy group and the Sepsis group.

### 2.3. Identification of EPCs

Cell surface markers CD133, CD34, CD31, and CD45 were detected by flow cytometry. FITC-UEA-I and Dil-AC-LDL staining were used to identify cells with good growth status. On the one hand, the cells were collected and resuspended with 200 *μ*L PBS. CD133 (bs-0395R-FITC, Bioss), CD34 (bs-0646R-FITC, Bioss), VEGFR2 (bs-0565R-FITC, Bioss), and CD45 (bs-0522R-FITC, Bioss) antibodies were added and incubated for 30 min away from light. Cells were washed with 1 mL PBS, which was repeated twice. The cells were suspended by adding 200 *μ*L PBS and then filtered by nylon mesh. The expression of CD133, CD34, CD31, and CD45 in the cells was detected by flow cytometry (A00-1-1102, Beckman, USA). On the other hand, the cells were inoculated into a 24-well plate preplaced with the slipper and cultured overnight. We discarded the medium, washed it with PBS for three times, added 250 *μ*L culture medium containing Dil-AC-LDL (10 *μ*g/mL) under dark conditions, and incubated for 4 h in an incubator with 5% CO_2_ at 37°C. After incubation, the medium was discarded, washed by PBS for three times, and fixed for 30 min with 4% paraformaldehyde. We discarded paraformaldehyde, washed with PBS for three times, added 250 *μ*L FUTC-UEA-1 (10 *μ*g/mL) at 37°C, and incubated in 5% CO_2_ for 1 h under dark conditions. Then, we washed with PBS for three times, added 250 *μ*L DAPI working solution (1 *μ*g/mL), and incubated in dark at 37°C for 10 min. Furthermore, we discarded the dye solution, washed with PBS for three times, and took images.

### 2.4. Cell Culture and Treatment

EPCs were cultured in a Dulbecco's Modified Eagle's Medium (DMEM) medium, 37°C, 5% CO_2_, saturated humidity incubator. They were divided into the Healthy group and the Sepsis group. To investigate the role of lncRNA IGF2-AS, we knocked down lncRNA IGF2-AS in EPCs of sepsis patients. They were divided into the si-NC group (EPCs of sepsis patients transfected si-NC) and the si-lncRNA IGF2-AS group (EPCs of sepsis patients transfected si-lncRNA IGF2-AS). Then, to investigate the role of HMGA1, we first knocked down HMGA1 in EPCs of sepsis patients. They were divided into two groups: si-NC group and si-HMGA1 group. Subsequently, we knocked down TYMS in EPCs of sepsis patients. The groups were the si-NC group and si-TYMS group. Next, lncRNA IGF2-AS was overexpressed in EPCs of sepsis patients. They were divided into the oe-NC group and the oe-lncRNA IGF2-AS group. After that, we gave different concentrations of dNTP treatment. They were grouped into oe-NC, oe-LncRNA IGF2-AS, oe-LncRNA IGF2-AS+1 *μ*M dNTP, oe-LncRNA IGF2-AS +10 *μ*M dNTP, and oe-LncRNA IGF2-AS+100 *μ*M dNTP. si-lncRNA IGF2-AS, si-HMGA1, and si-TYMS were synthesized by Sangon Biotechnology Co., Ltd. (Shanghai, China), and corresponding negative control si-NC was used to knock down lncRNA IGF2-AS, HMGA1, and TYMS. In order to overexpress lncRNA IGF2-AS, lncRNA IGF2-AS sequence was linked to LV003 vector. According to the instructions, vectors were transfected into EPCs of sepsis patients using lipofectamine 3000 reagent.

### 2.5. Quantitative Real-Time PCR (qRT-PCR)

The levels of lncRNA IGF2-AS, HMGA1, TYMS, ASC, Caspase-1, NLRP3, IL-18, RRM2, and TK1 were detected by qRT-PCR. Total RNA was extracted by Trizol method. The mRNA reverse transcription kit (CW2569) was purchased from Beijing ComWin Biotech. cDNA was obtained by reverse transcription using total mRNA of cells as template. By querying NCBI, primers of target genes were designed by the Primer5 software and biosynthesized by Sangon Biotechnology Co., Ltd. (Shanghai, China). A PCR reaction system containing UltraSYBR Mixture (CW2601, Beijing ComWin Biotech) was formulated. Then, fluorescence quantitative PCR instrument (QuantStudio1, Thermo) was used for PCR reaction and real-time monitoring of fluorescence signal. Finally, with *β*-actin as an internal reference, the relative expression levels of target genes in each group were calculated by 2^-*ΔΔ*Ct^ method. The primer sequences used in this study are shown in Table 2.

### 2.6. Western Blot (WB)

Total protein was extracted from EPCs using RIPA lysis buffer (#P0013B, Beyotime Biotechnology) according to the instructions. Protein was quantified in each group according to BCA protein assay kit. SDS-PAGE loading buffer (#MB2479, Meilunbio) was mixed. The mixture was heated in a boiling water bath at 100°C for 5 minutes. The protein was adsorbed onto PVDF membrane by gel electrophoresis. Primary antibodies against TSG101 (14497-1-AP, 1 : 1000, Proteintech), CD81 (66866-1-Ig, 1 : 1000, Proteintech), CD63 (25682-1-AP, 1 : 1000, Proteintech), ASC (10500-1-AP, 1 : 2000, Proteintech), Caspase-1 (#4199, 1 : 1000, CST), NLRP3 (19771-1-AP, 1 : 1000, Proteintech), IL-18 (10663-1-AP, 1 : 2000, Proteintech), HMGA1 (ab252930, 1 : 1000, Abcam), RRM2 (11661-1-AP, 1 : 2000, Proteintech), TK1 (15691-1-AP, 1 : 1000, Proteintech), TYMS (15047-1-AP, 1 : 1000, Proteintech), and *β*-actin (66009-1-IG, 1: 1000, Proteintech) were incubated at 4°C overnight. TBST was washed three times at room temperature and incubated with secondary HRP goat anti-mouse IgG (SA00001-1, 1 : 5000, Proteintech) and HRP goat anti-Rabbit IgG (SA00001-2, 1: 6000, Proteintech). ECL chromogenic exposure was performed. Odyssey Infrared Imaging System (Li-COR Biosciences, Lincoln, NE, USA) was applied to detect protein bands, and *β*-actin was used as an internal reference to detect expression levels.

### 2.7. EdU Assay

Cells were labeled with 50 *μ*M EdU (RiboBio, Guangzhou, China) and incubated with EPCs overnight. The cells were then fixed with 4% paraformaldehyde and glycine solution. The cells were incubated with the osmotic agent on a shaker for 10 minutes. Finally, cells were stained with 1× Apollo® and 1× Hoechst33342 working solution at dark and room temperature for 30 min, respectively. Cells were observed immediately after staining using an inverted biological microscope (DSZ2000X, Cnmicro).

### 2.8. Cell Counting Kit 8 (CCK-8) Assay

EPCs were digested with trypsin digestion solution to prepare cell suspension, which was inoculated into 96-well plates according to the ratio of 5 × 10^3^. Each group was set with three multiple wells for corresponding intervention treatment according to experimental groups and cultured for 24 h. After corresponding time of culture, the medium was discarded and replaced with 10 *μ*L CCK-8 working solution. The medium was incubated for 4 h in an incubator with 5% CO_2_ at 37°C. The absorbance was measured at 450 nm using Elx800 (BioTek, Winooski, Vermont, USA). The proliferation ability of cells was detected at 24, 48, and 72 h.

### 2.9. Immunofluorescence (IF)

The expression of Caspase-1 was detected by IF. We took out the slices, washed them with PBS 2-3 times, fixed with 4% paraformaldehyde for 30 min, and washed them with PBS 3 times, 5 minutes each time. Then, they were permeabilized with 0.5% TritonX-100 at 37°C for 30 minutes. After washing with PBS, 5% BSA was sealed at 37°C for 1 h; Caspase-1 primary antibody was incubated overnight at 4°C and washed with PBS for 3 times, 5 min each. Diluted fluorescent secondary antibody CoraLite594-conjugated Goat Anti-Rabbit IgG (SA00013-4, 1 : 200, Proteintech) was added. The cells were incubated at 37°C for 90 min and washed with PBS for 5 min, 3 times. DAPI working solution (Wellbio, China) was stained at 37°C for 10 min and washed with PBS for 5 min, three times. The tablets were sealed with buffered glycerin and observed under fluorescence microscope.

### 2.10. Transmission Electron Microscope (TEM)

EPCs were collected into a 10 mL centrifuge tube and then placed in a supercooled centrifuge at 4°C for 10 min. The supernatant was discarded, cleaned with PBS twice. 2.5% glutaraldehyde fixative solution was added overnight at 4°C, and the cells were rinsed with 0.1 M phosphoric acid rinse solution three times for 15 min. They were fixed with 1% osmium fixation solution for 2-3 h and rinsed with 0.1 M phosphoric acid rinse solution for 15 min three times. Then, they were treated with ethanol and acetone and encapsulated at room temperature. Ultrathin microtome was used for section, 3% uranium acetate-lead citrate was applied for double staining, and TEM (Opton, China) was used for observation.

### 2.11. Bioinformatics Prediction and RNA Immunoprecipitation (RIP) Assay

Starbase (http://starbase.sysu.edu.cn/index.php) predicted the binding site of lncRNA IGF2-AS to HMGA1. The binding of lncRNA IGF2-AS to HMGA1 was validated using the EZMagna RIP kit (Millipore, Massachusetts, USA) according to the manufacturer's specifications. To put it simply, EPCs from sepsis patients were dissolved in RIP lysis buffer at 4°C for 30 min and then incubated with RIP buffer containing magnetic beads. The beads were conjugated gated antibodies against Ago2 (CST, Boston, USA) or anti-Rabbit IgG (negative control, CST, Boston, USA). The precipitated RNA was analyzed by qRT-PCR, with total RNA as the input controls.

### 2.12. Co-immunoprecipitation (Co-IP)

Pretreated cells were rinsed once with precooled PBS and lysed with 600 *μ*L of IP lysis buffer on ice for 30 min. Then they were centrifugated at 12000 rpm for 15 min. The centrifuged supernatant was transferred to a new 1.5 mL centrifuge tube. Protein supernatant was divided into 3 tubules, which were whole protein, IgG, and IP, respectively. Supernatants were incubated with antibodies at 4°C overnight on a rotator. Then, we added 20 *μ*L prewashed protein A/G agarose beads for another 2 h. After extensive washing with a diluted lysis buffer, the lysate was used for western blot analysis.

### 2.13. Detection of dNTP Content

Gas chromatography time-of-flight mass spectrometry (GC-TOFMS) was used to determine the changes of dNTP in EPCs of sepsis patients. They were divided into two groups: oe-NC group and oe-lncRNA IGF2-AS group. Briefly, Acclaim C30 column with long carbon chain was used as analysis column, and gradient elution was performed with 20 mmol/L ammonium acetate and acetonitrile as mobile phases, at a flow rate of 0.4 mL/min, at 15°C and UV detection wavelength of 260 nm. Electrospray ionization was performed in positive ion mode. Reaction monitoring mode was selected for detection, and external standard method was used for quantitative analysis.

### 2.14. Isolation, Identification, and Transfection of Exosomes

According to the instructions of exosome extraction kit (EXOQ5A-1, SBI Company), exosomes secreted by bone marrow MSCs (HUM-iCell-s011, iCell, China) were extracted. Supernatants of MSCs were collected, and they were centrifuged at 3000 g for 10 min. The cells and cell fragments were discarded. The supernatant was transferred to a new centrifuge tube, and exosome extraction reagent was added to the cell supernatant in the ratio of EXOQ5A-1 = 5 : 1 by volume, and then mixed, and stood overnight at 4°C. Then, they were centrifuged at 1500 g for 30 min; the supernatant was discarded and centrifuged at 1500 g for 5 min. We carefully discarded the supernatant, precipitate as exosome, added 100 *μ*L PBS to resuspended precipitate. Exosomes were obtained for subsequent detection. They were divided into two groups: the supernatant group (negative control) and the Exosome group (MSC-derived exosomes). Subsequently, isolated and identified exosomes were used to treat EPCs from sepsis patients and were grouped into the EPCs group and the EPCs+exosomes group.

### 2.15. Nanoparticle Tracking Analysis (NTA)

The particle size, concentration, and distribution of NTA were analyzed by Particle Metrix. NanoSight NS300 was used to detect exosomes at 405 nm, and five videos of 60 s were collected for each sample. The data were analyzed by the NTA 3.0 software, and the Stokes-Einstein equation was used to calculate the fluid dynamics diameter of each exosome.

### 2.16. Characterization of Exosome Morphology

We soaked 10 *μ*L exosome suspension on copper wire and let it stand for 1-5 min in the oven at room temperature or 37°C. After the excess exosome samples were absorbed by filter paper, 10 *μ*L of phosphotungstic acid solution (about 1% concentration prepared by phosphoric acid buffer) was added to the prepared copper net and stood for 1-2 min. Then, the filter paper was absorbed to dry the excess dye on the copper net. Pure water was added to the copper net and then absorbed with filter paper, repeated two or three times to wash the excess phosphotungstic acid, then stood and dried, and observed the morphology of exosomes under TEM.

### 2.17. Exosome Uptake Experiment

Sterile slides were placed in the cell culture plate and inoculated with 2 × 10^4^/mL EPCs in the 12-well plate pre-placed with slides and maintained overnight in 1640 medium with 2% FBS. 20 *μ*g exosomes solution was resuspended with 250 *μ*L Diluent C solution. To prepare PKH-67 dye solution, exosomes solution was mixed with PKH-67 dye solution and incubated for 4 min. We added 420 *μ*L 10% BSA to bind the excess PKH-67 dye, added 1/5 volume of Exoquick-TC solution, mixed upside down, and kept upright at 4°C overnight. Centrifugation at 1500 g for 30 min, supernatant was discarded, and the precipitate was resuspended with 50 *μ*L PBS. PKH-67 labeled exosomes were added and co-incubated with EPCs for 6 h. The slides were removed and washed with PBS twice, fixed with 4% paraformaldehyde for 30 min, and washed with PBS for three times. The peptide was diluted with PBS to the working solution (1 : 100 dilution) and incubated for 30 min in dark, then washed with PBS for three times. Then, we added 1 mL 1 *μ*g/mL DAPI and incubated for 10 min at 37°C in dark, washed with PBS for three times, and observed under fluorescence microscope (Olympus, Japan).

### 2.18. Statistical Analysis

SPSS 20.0 was used for statistical analysis. Data were expressed in terms of mean ± standard deviation (mean ± SD). The student's *t*-test was used to analyze differences between two groups, and one-way analysis of variance (ANOVA) was utilized to compare data differences between multiple groups. The correlation between HMGA1 and TYMS was analyzed by Pearson Correlation Coefficient. *P* value < 0.05 was considered statistically significant.

## 3. Results

### 3.1. lncRNA IGF2-AS Affected the Activity of EPCs in Sepsis Patients

**Figure 1:**
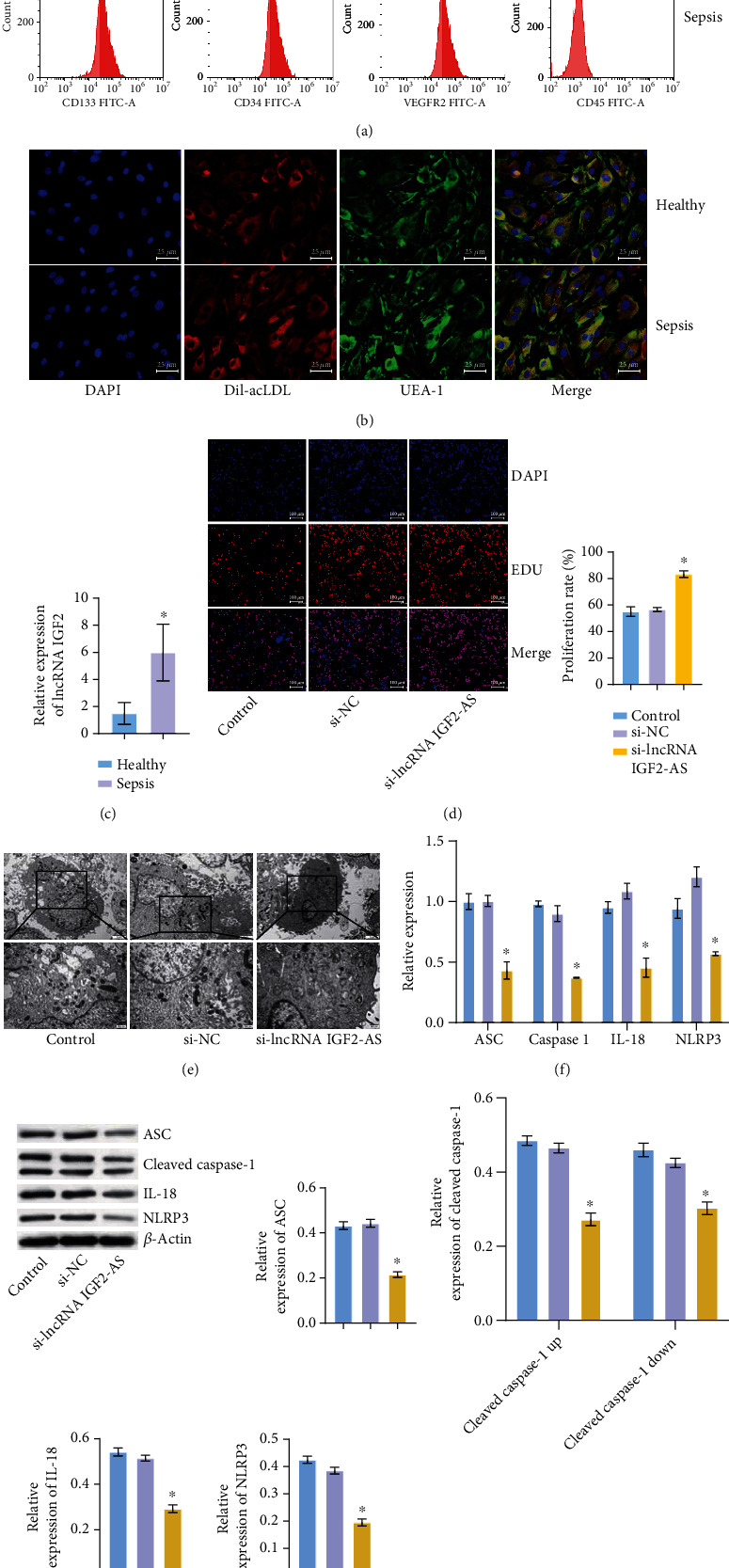
lncRNA IGF2-AS affected the activity of EPCs in sepsis patients. (a) Cell surface markers CD133, CD34, CD31, and CD45 were detected by flow cytometry. (b) IF was performed to identify EPCs in the Healthy and Sepsis groups. (c) qRT-PCR was applied to detect lncRNA IGF2-AS expression in EPCs of sepsis patients. (d) EdU was used to detect the proliferation of EPCs. (e) Pyroptosis of EPCs was detected by TEM. (f, g) qRT-PCR and WB were utilized to measure pyroptosis-related factors ASC, Caspase-1, NLRP3, and IL-18 expressions. ^∗^*P* < 0.05 vs. Healthy or si-NC.

Table 1 showed the clinical characteristic of sepsis patients and healthy subjects. Among them, the temperature, heart rate, lactate, and SOFA score were significantly increased in sepsis patients compared with healthy subjects, while PaO_2_/FiO_2_ was decreased in sepsis patients. Then, we isolated EPCs from the Healthy and Sepsis groups. To identify cells as EPCs, flow cytometry was used to detect EPCs surface markers, and the percentage of CD133, CD34, and CD31 in the Healthy and Sepsis groups was higher than 70%, 65%, and 60%, respectively. The expression level of CD45 was very low, only about 0.01% (Figure 1(a)). In addition, IF showed that cell membrane-bound to UEA-1 antibody was green, cell uptake of Dil-ac-LDL was red, and cell nuclear staining was blue after uptake of DAPI. Fusion image showed that cells with orange membrane changes were normally differentiated EPCs (Figure 1(b)). This indicated that EPCs in the Healthy group and the Sepsis group were successfully separated and identified. Next, we detected the expression of lncRNA IGF2-AS in EPCs. As shown in Figure 1(c), compared with the Healthy group, lncRNA IGF2-AS was highly expressed in EPCs of sepsis patients. We also conducted cell experiments to detect the function of EPCs. Compared with the si-NC group, the si-lncRNA IGF2-AS group had increased proliferation ability and decreased pyroptosis, which showed decreased cell swelling and pyroptosis body. In addition, the expressions of pyroptosis-related factors ASC, Caspase-1, NLRP3, and IL-18 were decreased (Figures 1(d)–1(g)). These results suggested that lncRNA IGF2-AS affected EPCs activity in sepsis patients.

### 3.2. lncRNA IGF2-AS Played a Regulatory Role by Binding HMGA1

**Figure 2:**
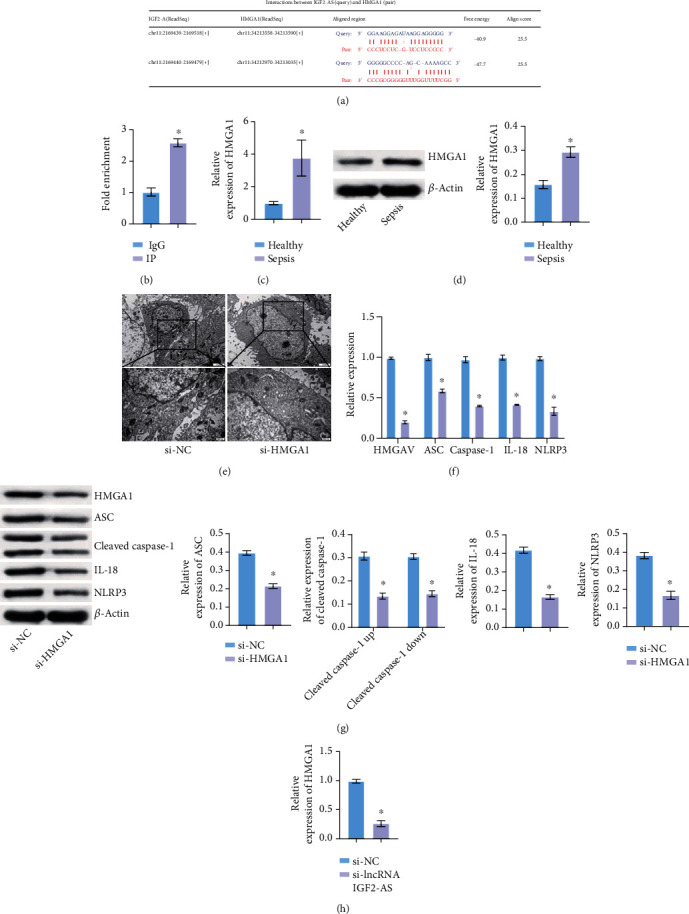
lncRNA IGF2-AS played a regulatory role by binding HMGA1. (a) Starbase predicted binding sites of lncRNA IGF2-AS to HMGA1. (b) RIP experiment verified the binding of lncRNA IGF2-AS to HMGA1. (c, d) qRT-PCR and WB were utilized to measure HMGA1 expression in EPCs of sepsis patients. (e) Pyroptosis of EPCs was detected by TEM. (f, g) qRT-PCR and WB were performed to verify HMGA1 interference efficiency and detect pyroptosis-related factors ASC, Caspase-1, NLRP3, and IL-18 expressions. (h) Expression of HMGA1 was detected by qRT-PCR. ^∗^*P* < 0.05 vs. Healthy or si-NC.

Starbase (http://starbase.sysu.edu.cn/index.php) showed that lncRNA IGF2-AS had binding sites with HMGA1 (Figure 2(a)). RIP experiment revealed that HMGA1 was the downstream potential binding protein of lncRNA IGF2-AS (Figure 2(b)). qRT-PCR and WB detection showed that HMGA1 was highly expressed in EPCs of sepsis patients compared with the Healthy group (Figures 2(c) and 2(d)). TEM showed that the pyroptosis of the si-HMGA1 group was reduced compared with the si-NC group (Figure 2(e)). Then, to study the role of HMGA1, we interfered with HMGA1. qRT-PCR and WB verified HMGA1 interference successfully (Figures 2(f) and 2(g)). qRT-PCR and WB showed that compared with the si-NC group, the levels of pyroptosis-related factors ASC, Caspase-1, NLRP3, and IL-18 were also decreased in the si-HMGA1 group (Figures 2(f) and 2(g)). In addition, compared with the si-NC group, HMGA1 expression in the si-lncRNA IGF2-AS group was reduced (Figure 2(h)). These results indicated that lncRNA IGF2-AS played a regulatory role by binding HMGA1.

### 3.3. Knocking Down lncRNA IGF2-AS Down-regulated dNTP Metabolization-Related Enzymes

**Figure 3:**
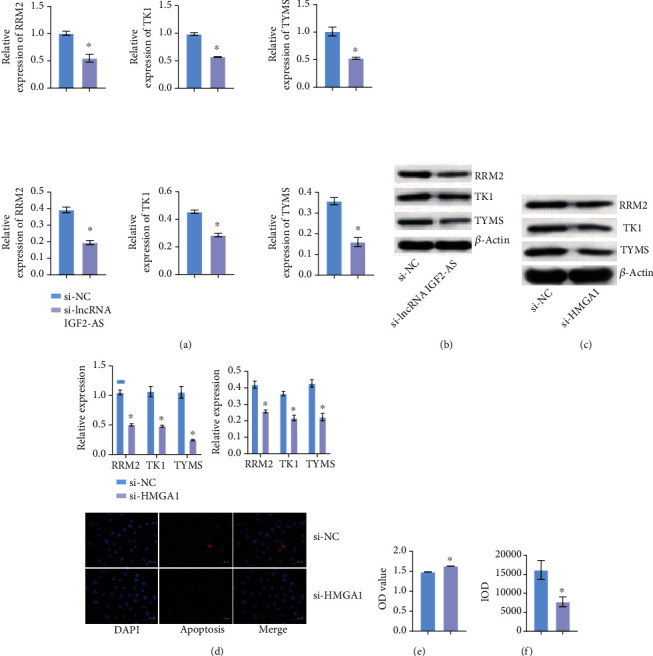
Knocking down lncRNA IGF2-AS down-regulated dNTP metabolization-related enzymes. (a, b) After knocking down lncRNA IGF2-AS, the expressions of RRM2, TK1, and TYMS were measured by qRT-PCR and WB. (c, d) After knocking down HMGA1, qRT-PCR, and WB was applied to detect expression of RRM2, TK1, and TYMS. (e) Proliferation of EPCs was detected by CCK-8 after knocking down HMGA1. (f) After knocking down HMGA1, IF was used to detect the expression of Caspase-1. ^∗^*P* < 0.05 vs. si-NC.

Next, we want to further explore lncRNA IGF2-AS effects on dNTP metabolism-related enzymes. As shown in Figures 3(a) and 3(b), compared with the si-NC group, the expression of RRM2, TK1, and TYMS decreased after si-lncRNA IGF2-AS interference. Compared with the si-NC group, expressions of RRM2, TK1, and TYMS were also decreased after si-HMGA1 interference (Figures 3(c) and 3(d)). We also tested cell functions after knocking down HMGA1, and CCK-8 showed that the proliferation of EPCs increased after si-HMGA1 interference (Figure 3(e)). IF staining for Caspase-1 showed that compared with the si-NC group, the positive expression of Caspase-1 decreased after knocking down HMGA1 (Figure 3(f)). These results suggested that knocking down lncRNA IGF2-AS down-regulated dNTP metabolization-related enzymes.

### 3.4. HMGA1 Was Related and Bound to TYMS

**Figure 4:**
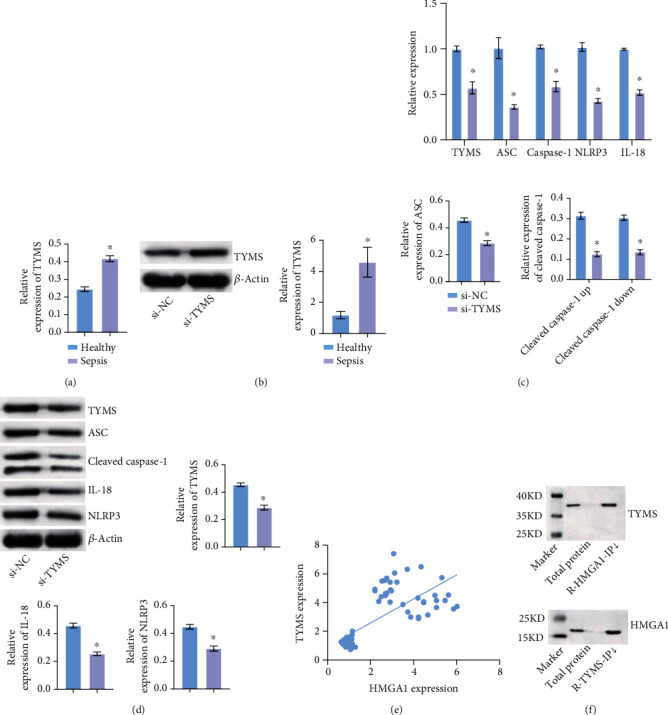
HMGA1 was related and bound to RRM2, TK1, and TYMS. (a, b) qRT-PCR and WB were applied to detect the expression of TYMS in EPCs of sepsis patients. (c, d) qRT-PCR and WB were performed to verify TYMS interference efficiency and measure the expression of pyroptosis-related factors ASC, Caspase-1, NLRP3, and IL-18. (e) Correlation Coefficient analysis between HMGA1 and TYMS. (f) Co-IP experiment demonstrated the binding of HMGA1 with TYMS. ^∗^*P* < 0.05 vs. Healthy or si-NC.

Next, we detected the expression of TYMS in EPCs. qRT-PCR and WB detection showed that TYMS was highly expressed in EPCs of sepsis patients compared with the Healthy group (Figures 4(a) and 4(b)). We then knocked down TYMS in the EPCs of sepsis patients. qRT-PCR and WB verified the success of TYMS interference (Figures 4(c) and 4(d)). qRT-PCR and WB showed that compared with the si-NC group, the expression levels of pyroptosis related factors ASC, Caspase-1, NLRP3, and IL-18 were decreased in the si-TYMS group (Figures 4(c) and 4(d)). Pearson Correlation Coefficient analysis showed that HMGA1 was positively correlated with TYMS (Figure 4(e)). Co-IP experiment showed that HMGA1 was combined with TYMS (Figure 4(f)). These results showed that HMGA1 was related and bound to TYMS.

### 3.5. Overexpression of lncRNA IGF2-AS Reduced the Level of dNTP, While dNTPs Could Save the Phenotype of lncRNA IGF2-AS

**Figure 5:**
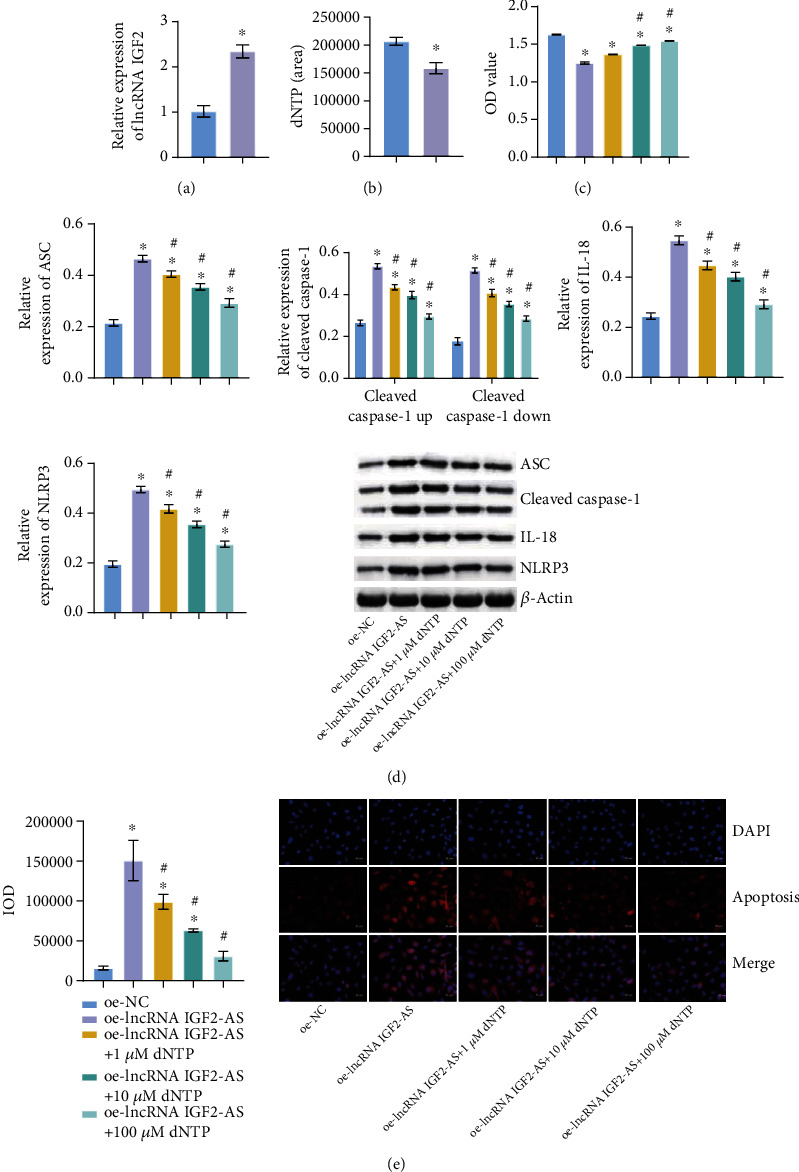
Overexpression of lncRNA IGF2-AS reduced the level of dNTP, while dNTPs could save the phenotype of lncRNA IGF2-AS. (a) qRT-PCR verification of lncRNA IGF2-AS overexpression efficiency. (b) dNTP content was measured by GC-TOFMS. (c) Proliferation of EPCs was detected by CCK-8. (d) qRT-PCR and WB were used to detect the expression of pyroptosis-related factors ASC, Caspase-1, NLRP3, and IL-18. (e) IF tested the expression of Caspase-1. ^∗^*P* < 0.05 vs. oe-NC, #*P* < 0.05 vs. oe-lncRNA IGF2-AS.

Then, lncRNA IGF2-AS was overexpressed in EPCs of sepsis patients. qRT-PCR verified the successful overexpression of lncRNA IGF2-AS (Figure 5(a)). After the content of dNTP was detected by GC-TOFMS, the level of dNTP decreased after the overexpression of lncRNA IGF2-AS (Figure 5(b)). After that, we gave different concentrations of dNTP treatment. CCK-8 showed that after overexpression of lncRNA IGF2-AS, the higher concentration of dNTP was given, the more cell proliferation was recovered (Figure 5(c)). qRT-PCR and WB showed that after overexpression of lncRNA IGF2-AS, the higher concentration of dNTP was given, the lower the expression levels of pyroptosis-related proteins ASC, Caspase-1, NLRP3, and IL-18 (Figure 5(d)). IF staining Caspase-1 showed that after overexpression of lncRNA IGF2-AS, the higher concentration of dNTP was given, the less positive Caspase-1 expression was, thus inhibiting pyroptosis (Figure 5(e)). These results indicated that overexpression of lncRNA IGF2-AS reduced the level of dNTP, while dNTPs could save the phenotype of lncRNA IGF2-AS.

### 3.6. MSC-Derived Exosomal lncRNA IGF2-AS Might Promote Pyroptosis of EPCs in Sepsis Patients

**Figure 6:**
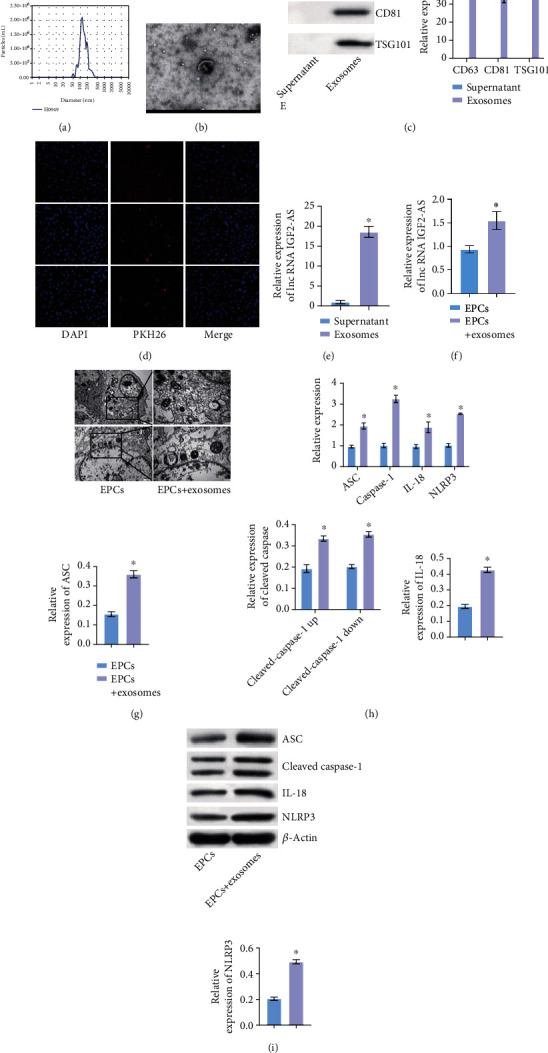
MSC-derived exosomal lncRNA IGF2-AS might promote pyroptosis of EPCs in sepsis patients. (a) Concentration and size detection of exosomes. (b) Exosome structure was observed by TEM. (c) Expression of exosome positive markers TSG101, CD81, and CD63 detected by WB. (d) Exosome uptake experiment. (e) Expression of lncRNA IGF2-AS in exosomes was detected by qRT-PCR. (f) qRT-PCR was used to detect the expression of lncRNA IGF2-AS in EPCs after exosome uptake. (g) Pyroptosis of EPCs was detected by TEM. (h, i) qRT-PCR and WB were performed to measure pyroptosis-related factors ASC, Caspase-1, NLRP3, and IL-18 expressions. ^∗^*P* < 0.05 vs. supernatant or EPCs.

We isolated exosomes derived from bone marrow MSCs. The concentration and size of exosomes were analyzed through Nanoparticle Tracking (Figure 6(a)). Under TEM, MSC-exosomes were observed to have circular and elliptical vesicle-like structures (Figure 6(b)). In addition, WB analysis showed that compared with the supernatant group, MSC-exosomes expressed TSG101, CD81, and CD63 (Figure 6(c)). Exosome uptake experiment showed MSC-exosomes was uptaken by EPCs (Figure 6(d)). qRT-PCR results showed that lncRNA IGF2-AS was expressed in MSC-exosomes compared with the supernatant group (Figure 6(e)). Then, we treated EPCs with MSC-exosomes to test the expression of lncRNA IGF2-AS and the function of EPCs. Compared with the EPC group, the expression of lncRNA IGF2-AS increased in the EPCs+exosomes group (Figure 6(f)). Compared with the EPCs group, the pyroptosis of the EPCs+exosomes group was increased, and the expressions of pyroptosis-related factors ASC, Caspase-1, NLRP3, and IL-18 were increased (Figures 6(g)–6(i)). These results revealed that MSC-derived exosomal lncRNA IGF2-AS might promote pyroptosis of EPCs in sepsis patients.

## 4. Discussion

Although the treatment of sepsis has developed rapidly in recent years, the incidence and mortality of sepsis are still increasing. Therefore, it is very important to find an effective means of diagnosis and treatment. In this study, we took EPCs of sepsis patients as the research object and explored lncRNA IGF2-AS and HMGA1 effects on pyroptosis of EPCs in sepsis patients and the involved mechanism through in vitro cell experiment. Our results suggested that lncRNA IGF2-AS regulated nucleotide metabolism by mediating HMGA1 to promote pyroptosis of EPCs in sepsis patients. In addition, MSC-derived exosomal lncRNA IGF2-AS may promote pyroptosis of EPCs in sepsis patients. Currently, there have been no reported studies on the related mechanisms of lncRNA IGF2-AS mediating HMGA1 in regulating nucleotide metabolism in sepsis, which is also the innovation of this study.

More and more evidences indicate that lncRNAs play a key regulatory role in sepsis. For example, Wang SM, et al. reported that lncRNA NEAT1 alleviated sepsis induced myocardial injury by regulating TLR2/NF-*κ*B signaling pathway [22]. Han D, et al. reported that knocking down lncRNA NKILA promoted cell viability in lipopolysaccharide-induced sepsis model by regulating miR-140-5p/CLDN2 axis and inhibited apoptosis, autophagy, and inflammation [23]. All of these studies indicate that lncRNAs play a key regulatory role in sepsis. IGF2, as a mitogen, is the first discovered parentally imprinted gene [24]. lncRNA IGF2-AS has been proved to play an important role in other diseases, including liver cancer [25], gastric cancer [26], and diabetic retinopathy [27], ect. However, there are still few reports on lncRNA IGF2-AS in sepsis. Our study found that, compared with the Healthy group, lncRNA IGF2-AS were highly expressed in the Sepsis group. Compared with the si-NC group, the si-lncRNA IGF2-AS group increased proliferation and decreased pyroptosis. This suggests that lncRNA IGF2-AS affects the activity of EPCs in sepsis patients. This is the first time that we reported the study on lncRNA IGF2-AS in sepsis.

HMGA1, as an architectural transcription factor, reshapes chromatin structure in different cancers and promotes the interaction between transcriptional regulatory proteins and DNA [28]. HMGA1 has been studied in sepsis. MSCs expressing a dominant-negative HMGA1 cells showed improved function during sepsis [16]. Grant MA, et al. reported that netropsin improves the survival rate of patients with endotoxemia by disrupting HMGA1 binding to NOS2 promoter [29]. At present, lncRNA has been reported to play a role in regulating HMGA1 in diseases. lncRNA GACAT3, as a competitive endogenous RNA of HMGA1, can alleviate cucurbitacin B-induced apoptosis in gastric cancer cells [30]. In addition, lncRNA ITGB2-AS1 promotes the progression of clear cell renal cell carcinoma by regulating miR-328-5p/HMGA1 axis [31]. However, studies on lncRNA IGF2-AS and HMGA1 in sepsis have not been reported. It was found that compared with the Healthy group, HMGA1 was highly expressed in the Sepsis group. Compared with the si-NC group, the proliferation ability of the si-HMGA1 group increased and pyroptosis decreased. In addition, compared with the si-NC group, the expression of HMGA1 in the si-lncRNA IGF2-AS group was decreased. This indicates that lncRNA IGF2-AS plays a regulatory role in sepsis by combining with HMGA1.

dNTPs are substrates for DNA synthesis [32]. Changes in dNTP pool are associated with increased mutations, genomic instability, and tumorigenesis [33]. Studies have shown that RRM2, TK1, and TYMS in cervical cancer tissues are significantly different from healthy tissues and are significantly correlated with overall survival in cervical cancer [34]. In addition, Wang H, et al. reported that mRNA levels of RRM2, TK1, and TYMS can predict lung cancer and other cancers [35]. Abnormal gene expression exists in children with idiopathic severe aplastic anemia, and RRM2, TTK, and TYMS in children's MSCs are significantly down-regulated [36]. Our study showed that, compared with the Healthy group, the expressions of TYMS were high in the Sepsis group. Compared with the si-NC group, the expression of RRM2, TK1, and TYMS in si-lncRNA IGF2-AS group was decreased. This suggests that knocking down lncRNA IGF2-AS down-regulates dNTP metabolization-related enzymes. In addition, HMGA1 is related and binds to RRM2, TK1, and TYMS. Compared with the si-NC group, pyroptosis in the si-TYMS group was reduced. Compared with the si-NC group, the expression of RRM2, TK1, and TYMS in the si-HMGA1 group was also decreased. This is also the first time that we reported the studies on RRM2, TK1, and TYMS in sepsis. In addition, in cancer, lncRNA lincNMR silencing can reduce dNTP level, while exogenous dNTPs can repair the proliferation defect caused by lincNMR deletion [19]. Our results are consistent with that. After overexpressing lncRNA IGF2-AS, dNTP level decreased, while the proliferation increased and pyroptosis decreased with higher concentration of dNTP.

The functional mechanism of MSCs has been the focus of research in recent years. More and more evidence supports the idea that MSCs function in paracrine mode [37]. To date, there have been over 200 preclinical studies of exosome-based therapies in many different animal models [38]. Although a growing number of studies have reported the therapeutic properties of MSC-derived exosomes, their potential mechanisms of action remain largely open questions. In sepsis, MSC-derived exosomes have also been studied. It has been reported that bone MSCs can alleviate sepsis induced lung injury and have effective immunomodulatory and immunosuppressive properties by secreting exosomes [39]. In addition, IL-1*β* activates exosome miR-21 secreted by MSCs to induce macrophage M2 polarization and improve sepsis [40]. In sepsis induced acute lung injury, MSc-derived exosomal lncRNA-P21 can protect epithelial cells in lipopolysaccharide-induced acute lung injury by sponging miR-181 [15]. In this study, MSC-derived exosomal lncRNA IGF2-AS also play a positive role in sepsis. We found that EPCs took up MSC-exosomes. Compared with the supernatant group, lncRNA IGF2-AS was expressed in the exosomes group. Compared with the EPCs group, the EPCs+ exosomes group had increased lncRNA IGF2-AS expression and increased pyroptosis. These results suggest that MSC-derived exosomal lncRNA IGF2-AS might promote pyroptosis of EPCs in sepsis patients. Exosomes are exogenous, and their secretion of lncRNA IGF2-AS causes appropriate pyroptosis, which plays a beneficial role in sepsis to a certain extent. We hypothesized that pyrophosis could resist bacterial infection during the initial stage of sepsis, because appropriate pyrophosis could reduce tissue damage. However, after moderate and severe sepsis, over activated pyrophosis could lead to septic shock or increase the risk of secondary infection. Therefore, combined with the beneficial effects of exosomes, this work needs to be verified by a large number of experiments.

In this paper, our study suggested that nucleotide metabolism in sepsis may be regulated by HMGA1. However, it is not clear whether the mesenchymal stem cell-derived exosomal lncRNA IGF2-AS regulated nucleotide metabolism by mediating HMGA1, which will be the focus of our future research. We know from clinical information about sepsis patients and healthy subjects, the temperature, heart rate, lactate, and SOFA score were significantly increased in sepsis patients compared with healthy subjects, while PaO_2_/FiO_2_ was decreased in sepsis patients. Furthermore, mesenchymal stem cell-derived exosomal lncRNA IGF2-AS might promote pyroptosis of EPCs in sepsis patients. These results suggested that nucleotide metabolism regulated by HMGA1 in sepsis and paracrine controlled exosome production may be related to the clinical conditions of sepsis patients and healthy subjects. However, the exact mechanism involved is not clear at present. In the future, we will explore the mechanism involved deeply.

In conclusion, in this study, we found that lncRNA IGF2-AS mediated HMGA1 to regulate nucleotide metabolism and promote pyroptosis of EPCs in sepsis patients. In addition, MSC-derived exosomal lncRNA IGF2-AS might promote pyroptosis of EPCs in sepsis patients. Our study provides important clues for identifying new therapeutic targets related to sepsis.

## Figures and Tables

**Table 1:** The clinical characteristic of sepsis patients and healthy subjects.

Characteristic	Sepsis patients (*n* = 12)	Healthy subjects (*n* = 12)	*P* value
Gender	6 males/6 females	6 males/6 females	None
Age (years)	60.2 ± 9.9	59.6 ± 9.4	0.884
BMI (kg/m^2^)	22.2 ± 2.1	21.8 ± 1.8	0.622
Temperature (°C)	37.4 ± 0.7	36.8 ± 0.3	0.007
Heart rate (beats/min)	97.4 ± 13.2	83.6 ± 8.1	0.005
PaO_2_/FiO_2_	305.8 ± 69.9	461.1 ± 11.4	<0.001
GCS	14.6 ± 0.9	15	0.123
SBP (mmHg)	121.9 ± 15.6	122.0 ± 11.2	0.988
MAP (mmHg)	74.8 ± 6.0	77.4 ± 5.1	0.267
Lactate (mmol/L)	2.2 ± 0.6	0.9 ± 0.2	<0.001
Platelet (×10^9^/L)	283.1 ± 68.3	262.1 ± 32.0	0.282
Bilirubin (*μ*mol/L)	15.0 ± 14.8	9.3 ± 4.0	0.208
Creatinine (*μ*mol/L)	91.8 ± 38.2	78.0 ± 13.1	0.248
SOFA score	2.1 ± 0.3	0	<0.001

BMI: body mass index; PaO2: alveolar oxygen partial pressure; FiO2: fractional inspired oxygen concentration; GCS: Glasgow coma scale; SBP: systolic blood pressure; MAP: mean arterial pressure; SOFA: sequential organ failure assessment. The clinical characteristics of sepsis patients and healthy subjects were shown as mean ± standard deviation (mean ± SD).

**Table 2:** The primer sequences used in this study.

Name	Primers
lncRNA IGF2-AS-F	CACCTTGTCAGGATCTGGGC
lncRNA IGF2-AS-R	GTCCAACAGAAGGGTCTCGG
ASC-F	GAACTGGACCTGCAAGGACT
ASC-R	AGCTGGCTTTTCGTATATTGTGTA
Caspase-1-F	AGGACAACCCAGCTATGCC
Caspase-1-R	TGGATAAATCTCTGCCGACT
NLRP3-F	GCCACGCTAATGATCGACT
NLRP3-R	TCTTCCTGGCATATCACAGT
IL-18-F	TCTTCATTGACCAAGGAAATCGG
IL-18-R	TCCGGGGTGCATTATCTCTAC
HMGA1-F	GCATCCCAGCCATCACTC
HMGA1-R	GCTTCTCAGTGCCGTCCTT
RRM2-F	GGCGCGGGAGATTTAAAGG
RRM2-R	CACGGAGGGAGAGCATAGTG
TK1-F	GGCACAGAGAAGGAGCAGATT
TK1-R	CAGAAGGCCAAGGTGTGGTC
TYMS-F	TGGAGGAGTTGCTGTGGTTT
TYMS-R	TGGTCAACTCCCTGTCCTGA
*β*-Actin-F	ACCCTGAAGTACCCCATCGAG
*β*-Actin-R	AGCACAGCCTGGATAGCAAC

## Data Availability

The data used to support the findings of this study are included within the article.
